# Supplementary material for: Attitudes toward the Legalization of Euthanasia or Physician-Assisted Suicide in South Korea: A Cross-Sectional Survey
Source: Int J Environ Res Public Health. 2022 Apr 24;19(9):5183. doi: 10.3390/ijerph19095183 (PMC9105789; doi:10.3390/ijerph19095183)
Supplement: Supplementary file 1 [file ijerph-19-05183-s001.zip › ijerph-1641664-supplementary.pdf]

Table S1. Respondents' reasons for agreeing or disagreeing with the legalization of EAS according to the intersection of sex x age

				Age (years), N (%)	
Reasons for agreement (N = 763)	Total N (%)	Sex	N (%)	20-49	≥50
Meaninglessness of the rest of life	235 (30.8)	Male	120 (31.8)	55 (27.4)	65 (36.9)
		Female	115 (29.8)	55 (27.4)	60 (32.3)
Right to a good death	198 (26.0)	Male	107 (28.4)	59 (29.4)	48 (27.3)
		Female	91 (23.6)	50 (25.0)	41 (22.0)
Alleviation of suffering	157 (20.6)	Male	67 (17.8)	41 (20.4)	26 (14.8)
		Female	90 (23.3)	51 (25.5)	39 (21.0)
Family suffering and burden	113 (14.8)	Male	50 (12.9)	25 (12.4)	25 (14.2)
		Female	63 (16.3)	26 (13.0)	37 (19.9)
Social burden due to medical expenses and care	35 (4.6)	Male	20 (5.3)	13 (6.5)	7 (4.0)
		Female	15 (3.9)	10 (5.0)	5 (2.7)
No violation of human rights	24 (3.1)	Male	13 (3.4)	8 (4.0)	5 (2.8)
		Female	11 (2.8)	7 (3.5)	4 (2.2)
Importance of the right to self-determination	1 (0.1)	Male	0 (0.0)	0 (0.0)	0 (0.0)
		Female	1 (0.3)	1 (0.5)	0 (0.0)
Reasons for disagreement (N = 237)	Total N (%)	Sex	N (%)	20-49	≥50
Respect for life	105 (44.3)	Male	47 (37.3)	28 (34.6)	19 (42.2)
		Female	58 (52.3)	31 (50.0)	27 (55.1)
Violation of the right to self-determination	37 (15.6)	Male	24 (19.0)	14 (17.3)	10 (22.2)
		Female	13 (11.7)	4 (6.5)	9 (18.4)
Risk of abuse or overuse	31 (13.1)	Male	15 (11.9)	9 (11.1)	6 (13.3)
		Female	16 (14.4)	8 (12.9)	8 (16.3)
Violation of human rights	29 (12.2)	Male	18 (14.3)	13 (16.0)	5 (11.1)
		Female	11 (9.9)	8 (12.9)	3 (6.1)
Risk of misdiagnosis	23 (9.7)	Male	15 (13.5)	12 (14.8)	3 (6.7)
		Female	8 (7.2)	8 (12.9)	0 (0)
Possibility of recovery	12 (5.1)	Male	7 (5.6)	5 (6.2)	2 (4.4)
		Female	5 (4.5)	3 (4.8)	2 (4.1)

EAS, euthanasia and physician-assisted suicide

Table S2. Univariate logistic regression analyses of factors related to the legalization of EAS

Factors	Agree vs. Disagree (Ref)	
	OR	95% CI
Age in years at survey		
<50 (Ref)	1	
≥50	1.216	0.906-1.631
Sex		
Male (Ref)	1	
Female	1.162	0.868-1.556
Educational background		
College graduate or post-graduate (Ref)	1	
HS graduate/GED or below	1.138	0.848-1.526
Monthly household incomes		
≥ $3,000 (Ref)	1	
< $3,000	1.08	0.757-1.541
Marital status		
Single/widowed/ divorced/ separated (Ref)	1	
Married/ living with a partner	1.18	0.859-1.620
Religion		
Religious (Ref)	1	
Nonreligious	1.016	0.751-1.377
Rural/Urban area		
Rural/suburban (Ref)	1	
Urban	1.28	0.953-1.720
Job status		
Occupied (Ref)	1	
None occupied	1.16	0.824-1.633
Comorbidity		
None (Ref)	1	
More than one	1.213	0.878-1.675
General Health Status		
≥Very good (Ref)	1	
<Very Good	1.281	0.940-1.746
Physical Health Status		
≥Very Good (Ref)	1	
<Very Good	1.397	1.041-1.875
Mental Health Status		
≥Very good (Ref)	1	
<Very Good	1.333	0.989-1.796
Social Health Status		
≥Very good (Ref)	1	
<Very Good	1.326	0.980-1.793
Spiritual Health Status		
≥Very good (Ref)	1	

<Very Good	1.188	0.869-1.624
Political Tendency		
Center (Ref)	1	
Progressive	1.147	0.793-1.659
Conservative	1.337	0.942-1.897

EAS, euthanasia and physician-assisted suicide; Ref, reference; OR, odds ratio; CI, confidence interval
